# Anesthesiologist to Patient Communication: A Systematic Review

**DOI:** 10.1001/jamanetworkopen.2020.23503

**Published:** 2020-11-12

**Authors:** Michael J. Tylee, Gordon D. Rubenfeld, Duminda Wijeysundera, Michael C. Sklar, Sajid Hussain, Neill K. J. Adhikari

**Affiliations:** 1 Department of Critical Care Medicine, Sunnybrook Health Sciences Centre, Toronto, Ontario, Canada; 2 Department of Anesthesia and Pain Management, University Health Network, Toronto General Hospital, Toronto, Ontario, Canada; 3 Department of Anesthesia, University of Toronto, Toronto, Ontario, Canada; 4 Interdepartmental Division of Critical Care, University of Toronto, Li Ka Shing Knowledge Institute, Toronto, Ontario, Canada; 5 Department of Anesthesia, St Michael’s Hospital, Toronto, Ontario, Canada; 6 Department of Intensive Care Medicine, King AbdulAziz Medical City, Riyadh, Saudi Arabia

## Abstract

**Importance:**

Many patients are admitted to the intensive care unit following surgery, and some of them will experience incomplete recovery. For patients in this situation, preoperative discussions regarding patient values and preferences may direct care decisions. Existing literature shows that it is uncommon for surgeons to have these conversations preoperatively; it is unclear whether anesthesia professionals engage with patients on this topic prior to surgery.

**Objective:**

To review the literature on communication between patients and anesthesia professionals, with a focus on discussions related to postoperative critical care.

**Evidence Review:**

MEDLINE and Web of Science were searched using specific search criteria from January 1980 to April 2020. Studies describing encounters between patients and anesthesia professionals were selected, and data regarding study objectives, study design, methodology, measures, outcomes, patient characteristics, and clinical setting were extracted and collated. The Preferred Reporting Items for Systematic Reviews and Meta-analyses (PRISMA) reporting guideline was followed.

**Findings:**

A total of 12 studies including 1284 individual patient encounters were eligible for inclusion in the review. These studies demonstrated that communication between patients and anesthesia professionals related to postoperative care is rare: only 2 studies reported communication regarding adverse postoperative events, and this communication behavior was reported in only 46 of 1284 consultations (3.6%) across all studies. Additional findings were that communication during these encounters is dominated by anesthetic planning and perioperative logistics, with variable discussion of perioperative risks vs benefits and infrequent elicitation of patient values and preferences. Some data suggest that patients wish to be involved in perioperative decision-making but are often limited by an incomplete understanding of risks and benefits.

**Conclusions and Relevance:**

This systematic review found that communication in anesthesia is dominated by anesthetic planning and discussion of preoperative logistics, whereas postoperative critical care is rarely discussed. Most patients who are admitted to an intensive care unit after a major operation will not have had a discussion regarding goals of care specific to protracted recovery or prolonged intensive care with their anesthesiologist.

## Key Points

**Question:**

Do anesthesiologists or other anesthesia professionals engage in discussions with patients regarding decisions with implications beyond the operating room?

**Findings:**

In this systematic review of the literature on communication between patients and anesthesia professionals, limited data were found on communication regarding perioperative decisions with implications that reach beyond the operating room. These data suggest that communication between patients and anesthesia professionals during preoperative encounters is dominated by discussion of anesthetic planning and perioperative logistics, with variable discussion of risks vs benefits and infrequent discussion of postoperative care or elicitation of patient values and preferences.

**Meaning:**

These findings suggest that patients who become critically ill following scheduled surgical interventions are unlikely to have had discussions with their anesthesiologist regarding values and preferences for navigating complex postoperative care decisions, such as prolonged invasive ventilation, protracted hospital stay with incomplete recovery, or end-of-life care.

## Teaser

This systematic review studies the literature on communication between patients and anesthesia professionals, with a focus on discussions related to postoperative critical care.

## Introduction

Communication with patients about therapeutic options and care plans is a critical component of shared decision-making and is particularly important when a decision may result in a major or permanent change in a patient’s health status. This situation is relatively common for patients undergoing major surgery. Surgeons and anesthesiologists are the principal clinicians with the opportunity and, arguably, the responsibility to elicit values and preferences about postoperative care from surgical patients to inform care decisions if patients become critically ill and lose decisional capacity postoperatively. Previous work suggests that surgeons uncommonly elicit patient preferences regarding postoperative critical illness preoperatively, even for high-risk patients.^1,2^ Anesthesiologists also have the opportunity to elicit patient values and preferences preoperatively, and some members of the specialty have an interest in expanding anesthesiologists’ role in perioperative medicine.^3,4,5^ Knowledge and communication of medical and surgical complications after surgery, as opposed to complications of the anesthetic, are essential to this role. However, the extent of anesthesiologists’ responsibility and their ability to perform this role is not clear, and there are likely variable professional expectations for patient-anesthesiologist communication in different health care systems and settings.

There are few data on communication during anesthesia consultations. Although studies on anesthesiologist-patient communication have been narratively reviewed,^6,7^ there is no systematic review on this topic. In this review, a systematic search strategy was used to extract and collate data on communication between anesthesia professionals and patients, and the methodological quality of existing studies was assessed. A synthesis of the data focused on communication about postoperative critical illness is presented.

## Methods

A systematic review of the literature on communication between anesthesia professionals and patients was performed to address the following question: in preoperative anesthetic encounters, what are the patterns and content of communication between anesthesia professionals and patients as evaluated by qualitative or mixed methods? Reporting is consistent with the Preferred Reporting Items for Systematic Reviews and Meta-analyses (PRISMA) reporting guideline.^8^

### Information Sources and Search

A MEDLINE search was performed (from 1980 to April 2020) to retrieve any studies with a focus on communication between patients and anesthesia professionals (eAppendix 1 in the Supplement). A 1-generation, forward-and-backward search on Web of Science was then performed using each of the included studies from the MEDLINE search to identify additional relevant studies.

### Study Eligibility, Selection, and Data Extraction

Only studies with data describing specific encounters between patients and anesthesia professionals were included. Studies with a primary focus other than communication, studies on communication during anesthesia procedures, and studies examining communication with children were excluded (see eAppendix 2 and eAppendix 3 in the Supplement). In addition, studies that developed or evaluated communication interventions were excluded because these studies prescribed communication strategies instead of evaluating established communication practices. The search was limited to studies published in English, which generally gives a sufficient assessment of a given topic,^9,10^ and to studies published after 1980. Three reviewers (M.J.T., S.H., and M.C.S.) performed title screening, and 1 reviewer (M.J.T.) retrieved the full text of relevant titles, selected studies, and extracted data.

### Methodological Quality Review

One reviewer (M.J.T) assessed the quality of all studies using the previously validated Critical Appraisal Skills Program (CASP) tool for Qualitative Studies.^11^ A second reviewer (N.K.J.A.) verified these assessments.

### Statistical Analysis

Individual study results and quality reviews are presented, and overall results are synthesized descriptively. Variables and outcomes extracted from individual studies were too diverse for quantitative synthesis. Continuous data are expressed as means with SDs or as medians with interquartile ranges (IQR). No statistical testing was conducted.

## Results

### Search and Study Selection

The Figure shows an overview of study selection. Thirty full-text articles from the search were reviewed, of which 20 studies were excluded (see eAppendix 4 in the Supplement). Seventeen of these studies were excluded because they did not include any data about anesthesiologist-patient communication during routine encounters. Three studies were excluded because they were about communication during procedures. The remaining 10 studies were included, and the Web of Science search returned 2 more studies, resulting in 12 studies for review.^12,13,14,15,16,17,18,19,20,21,22,23^

**Figure.**
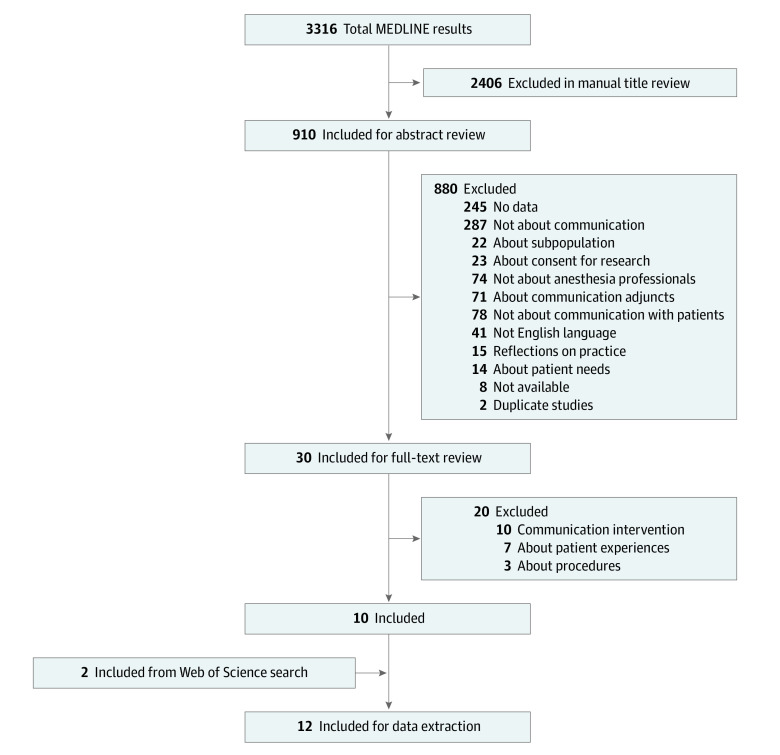
Study Selection

### Study Characteristics

Study characteristics are summarized in Table 1. All studies included descriptive statistics, and 5 studies^13,14,15,20,23^ performed some statistical modeling. Ten studies collected raw communication data on clinical encounters by audiotaping,^15,17,18,19,20,22,23^ videotaping,^16^ or direct observation with an experienced observer.^12,13^ One study collected data using questionnaires only,^14^ and another used semistructured interviews.^21^ Of the studies that performed qualitative analyses, only 1 study^15^ specified a qualitative analysis approach and framework^24^ for data coding.

**Table 1.** Study Characteristics

Source	Setting and patients	Objective	Study design and methodology	Data type and analysis
Babitu and Cyna,^12^ 2000	68 female patients enrolled following preanesthesia assessment in Australia. No risk stratification data of patients provided.	Determine whether patients understood technical terms used in preanesthesia assessment.	Observational study with standardized patient questionnaire.	Direct observation with expert observers. Number of technical terms used per consultation. Number and nature of technical terms not understood by patients. Descriptive statistics.
Barneschi et al,^13^ 2002	Preoperative assessments of 272 patients for elective general surgery in Italy. Patients for elective surgery; mostly ASA class I or II (n = 224) with some ASA class III patients (n = 31).	Determine how many patients received information about risks of anesthesia during preoperative consultations with and without priming using an information pamphlet.	Observational study with standardized patient questionnaires.	Direct observation with experienced observers. Number of anesthesia complications discussed preoperatively. Descriptive statistics.
Flierler et al,^14^ 2013	197 Preoperative patients undergoing elective surgery with options for anesthesia (ie, general or regional) in Switzerland. Mostly ASA class I or II (n = 177) with some ASA class III patients (n = 20).	Assess congruence between patient and health care professional perception of patient preferences and comparison to anesthetic option ultimately chosen.	Quantitative observational study. Survey-based study in a defined population selected based on convenience.	Survey scores. Multiple regression model for determining association between socioeconomic factors and desired level of involvement in decisions.
Gentry et al,^15^ 2017	97 Parents of children undergoing elective noncardiac surgery. No risk stratification data of patients provided.	Comprehensively characterize the informed consent discussion.	Quantitative observational study. Audio-taping of real patient interviews with patients and health care professionals.	Audio recording of consent conversations with subsequent coding and quantification of specific elements. Survey data evaluating demographic characteristics and subjective satisfaction levels. Logistic regression to evaluate associations between consent elements and parental recall.
Kindler et al,^16^ 2005	57 Patient encounters in preadmission clinic in Switzerland. No risk stratification data of patients provided.	Describe the nature of the patient-anesthetist interaction.	Semiqualitative observational study. Videotaping of real patient interviews with patients and health care professionals masked to study aims.	Raw data video footage of interactions. Utterances from the patient and anesthetist were coded using a previously validated coding tool. Association of frequency of specific utterances with patient involvement assessed by Pearson product-moment correlation.
Lagana et al,^17^ 2012	91 Patients and their parents or guardians on the day of surgery in Australia. Mostly ASA class I or II (n = 88) with some ASA class III patients (n = 12). Note that not all patient enrolled had complete data for analysis.	Observe and identify the number and nature of anesthesia risks considered and communicated to parents/guardians.	Semiqualitative observational study. Audio-taping of real patient interviews with patients and health care professionals masked to study aims.	Audio-recorded transcripts. Number of risks and discussion of risks identified from transcripts by 2 separate researchers. Descriptive statistics.
Nuebling et al,^18^ 2004	57 Patient encounters in preadmission clinic in Switzerland. No risk stratification data of patients provided.	Observe the association between physicians’ reassuring utterances with a variety of patient utterances.	Semiqualitative observational study. Audio-taping of real patient interviews.	Audio-recorded transcripts. Utterances from the patient and anesthetist were coded using a previously validated coding tool. Assessment of probability of reassuring utterances from the physician based on prior utterances by the patient.
Sandberg et al,^19^ 2008	26 Consultations in preoperative clinic in the United States. No risk stratification data of patients provided.	Quantify the amount of information given by anesthesia clinicians during preanesthetic interviews.	Semiquantitative observational study.	Audio-recorded transcripts. Descriptive statistics.
Stubenrouch et al,^20^ 2017	80 Patients undergoing elective procedures with at least 3 options for anesthesia (general, neuraxial, regional). No risk stratification data of patients provided.	Determine the level of shared decision-making in anesthesia consultations.	Quantitative observational study. Shared decision-making measured by OPTION score; patient and physician subjective assessments with SDM-Q-9 survey.	Survey scores. Descriptive statistics. Multiple regression models to determine associations between degree of shared decision-making and satisfaction with care.
Tait et al,^21^ 2011	263 Parents interviewed while their child was in surgery in Michigan. No risk stratification data of patients provided.	Examine the information that parents seek regarding their child’s anesthesia, what they are told, who told them, and how much of the information they recall.	Mixed quantitative and qualitative observational study.	Semistructured interview plus survey. Descriptive statistics and χ^2^ test to determine the association, if any, between who and when consent was taken, and volume of information recalled.
Trumble et al,^22^ 2015	14 Patients having epidural catheters inserted in Australia. No risk stratification data of patients provided.	Describe and quantify the risks and benefits of epidural anesthesia during the consent process.	Qualitative observational study.	Audio-recorded transcripts. Descriptive statistics.
Zollo et al,^23^ 2009	27 Interviews with standardized patients in preanesthesia clinic in New York. No risk stratification data of standardized patients provided.	Observe and describe the patterns of communication in the preanesthesia clinic with 2 types of standardized patients.	Quantitative observational study with standardized patients.	Audio-recorded transcripts. Descriptive statistics.

Abbreviations: ASA, American Society of Anesthesiologists; OPTION, Observing Patient Involvement Scores; SDM-Q-9, 9-item Shared Decision-Making Questionnaire.

### Methodological Quality

The summary of the methodological quality is shown in Table 2. Only 4 studies^14,16,18,20^ used previously validated tools to collect or code data, and 1 study^21^ created and validated a survey. Eight studies^12,15,16,17,18,19,20,22^ used 2 or more assessors to code recorded data. Eleven studies^12,13,15,16,17,18,19,20,21,22,23^ were evaluated on all CASP criteria, with a median (IQR) score of 4 of 5 (3-5). One study^14^ was only evaluated on 4 of the CASP criteria and scored 3 of 4. Methodological issues and assessment of quantitative analyses for studies that conducted statistical modeling are shown in Table 3.

**Table 2.** Critical Appraisal Skills Program Tool Scoring and Quality of Evidence

Source	Clear objective	Appropriate methodology	Data collection appropriate	Validated tools	Multiple assessors	Quality of evidence^a^
Babitu and Cyna,^12^ 2000	Yes	Yes	No	No	Yes (not during data coding)	4
Barneschi et al,^13^ 2002	Multiple Objectives	Only for descriptive objectives	Yes	No	No	4
Flierler et al,^14^ 2013	No clear primary objective	Only for descriptive objectives	Yes	Yes	NA	3
Gentry et al,^15^ 2017	Yes	Only for descriptive objectives	Yes	No	Yes	4
Kindler et al,^16^ 2005	Yes	Yes	Yes	Yes	Yes	4
Lagana et al,^17^ 2012	Yes	Yes	Yes	No	Yes	4
Nuebling et al,^18^ 2004	Yes	Yes	Yes	Yes	Yes	4
Sandberg et al,^19^ 2008	Yes	Yes	Yes	No	Yes	4
Stubenrouch et al,^20^ 2017	Yes	Only for descriptive objectives	Yes	Yes	Yes	4
Tait et al,^21^ 2011	No clear primary objective	No	Yes	Validated during study (data not shown)	Not clear	4
Trumble et al,^22^ 2015	Yes	Yes	Yes	No	Yes	4
Zollo et al,^23^ 2009	Yes	Yes	Yes	No	Not clear	4

Abbreviation: NA, not applicable.

^a^ Quality of evidence follows rating scheme from Oxford Centre for Evidence Based Medicine.

**Table 3.** Methodological Issues Identified in Included Papers

Source	Potential for bias
Babitu and Cyna,^12^ 2000	Hawthorne effects: patients primed to think about technical terms by enrollment; presence of observer biases anesthesiologist. May exclude terms not on standardized list used in the study. Small sample and limited patient population.
Barneschi et al,^13^ 2002	Small sample. Tools not validated; data only assessed and coded by 1 person. High chance of Hawthorne effect with a direct observer. Data about risks discussed was limited to predetermined list of potential risks. Large proportion of patients had previous anesthesia, which may have reduced the chance the anesthesiologist would discuss risks. Quantitative analysis: no primary outcome, no adjustment for multiple comparisons, no information on logistical model variable selection or assessment of modeling assumptions. Full final model not presented in the article.
Flierler et al,^14^ 2013	Not designed around a primary objective. Quantitative analysis: no primary outcome, poor justification for sample size.
Gentry et al,^15^ 2017	Small sample of consecutively enrolled patients. Measures of parental recall and understanding of information in the pediatric preanesthetic encounter were based solely on the perspective/opinion of the parent, with no objective assessment. Tools for variable assessment not validated. Quantitative analysis: 2 primary outcomes, no sample size calculation, not clear how clustering by clinician was included into model.
Kindler et al,^16^ 2005	No major sources of bias identified beyond selection bias; Hawthorne effect minimized well.
Lagana et al,^17^ 2012	Substantial potential for Hawthorne effect given the direct proximity of observer and the nature of the research question. Did not use validated tool for data collection.
Nuebling et al,^18^ 2004	Large potential for Hawthorne effect.
Sandberg et al,^19^ 2008	Likely underreported unexplained medical terms used in consultations, given that this is defined by patient queries and these are likely to be a subset of the medical terms misunderstood by patients.
Stubenrouch et al,^20^ 2017	Large potential selection bias. Quantitative analysis: arbitrary exclusion of physicians with low representation in data set, variable selection in multivariable model not explained or justified, no assessment of modeling assumptions.
Tait et al,^21^ 2011	Entire data set is from parental recall, thus, high risk of recall bias. Selection bias of parents willing to participate, although enrollment was 89% successful.
Trumble et al,^22^ 2015	Large potential for Hawthorne effect. Selection bias of patient and anesthesiologists willing to participate. Small sample size.
Zollo et al,^23^ 2009	Substantial potential for Hawthorne effect given that the participants knew the interviews were conducted with standardized patients. Standardized patient roles may represent outliers from general population. Quantitative analysis: unclear which specific variables were the dependent variables in multivariable modeling (ie, no clear hypothesis or association under evaluation), no justification of covariates included in models, no assessment of modeling assumptions or multicollinearity.

### Description of Content of Communication

Only 2 studies reported communication regarding adverse postoperative medical events, and this communication behavior was reported in only 46 of 1284 consultations (3.6%) across all studies. An a priori decision was made to specifically evaluate papers for communication data in the following categories: (1) discussion of therapeutic options including informed consent, patient comprehension, and risks/benefits, (2) elicitation of values and preferences, (3) shared decision-making, and (4) communication about postoperative care. These categories were chosen because they highlight communication that is central to patient-physician consultations around major interventions. Because of the broad types of data found in the review, the second and third categories were collapsed into a single category, and other data was added as a category to capture data that did not fit into previously defined categories. Study results are summarized in Table 4.

**Table 4.** Summary of Study Results

Source	Objective	Measures	Consent and patient comprehension	Shared decision-making	Discussion of postoperative care	Other data
Babitu and Cyna,^12^ 2000	Determine whether patients understood technical terms used in preanesthesia assessment.	No. of technical terms used in consultations; No. of technical terms not understood by patients.	89.9% of the technical terms used by anesthesiologists were understood by patients. Patients failed to understand ≥1 of the terms used by the anesthesiologist in 47% of consultations.	No data.	No data.	No additional data.
Barneschi et al,^13^ 2002	Determine how many patients received information about risks of anesthesia during preoperative consultations with and without priming using an information pamphlet.	No. and type of anesthetic risks discussed; Patient understanding of risk; Patient satisfaction scores.	Without patient priming, only 44% of assessments included disclosure and discussion of anesthetic risks. When the anesthesiologist discussed risks, >95% patients were satisfied with the discussion. When no risks were discussed, >80% of patients believed that there were no risks from anesthesia. Among those who feared the risks of anesthesia and were given no information about risk, more than half would have preferred to have a discussion of risks.	No data.	Death or severe permanent harm discussed in 20/272 (7.4%) and 22/272 (8.1%) of interviews, respectively. Postoperative pain mentioned in 36 interviews (13.2%).	Addition of a patient primer with a questionnaire focused on perioperative risks increases the chances that risks are discussed during the preoperative assessment.
Flierler et al,^14^ 2013	To assess patients’ preferences on being involved in shared decision-making and its influence on their satisfaction.	Patient and health care professional perceptions of ideal and actual level of patient involvement in decision-making; Patient and health care professional perceptions that specific items on a list of shared decision-making components were completed during encounters; Patient satisfaction scores.	No data.	Overall, >90% of patients wished to be involved in decisions about care. Good concordance between anesthetists and patient’s perceptions of desired patient involvement and actual patient involvement in perioperative decisions. Anesthetists tended to underestimate patients’ desire for shared decision-making. Patients believed that they understood benefits and drawbacks to each anesthetic option 92% of the time, while anesthetists believed this was true only 69% of the time.	No data.	Patient satisfaction scores were weakly correlated with patient desire to be involved in decision-making but were not affected by concordance between patient and anesthesiologist perception of patients’ desire to be involved in decision-making. In a multivariable model, the degree of shared decision-making and patient age were the only variables that were associated with patient satisfaction scores.
Gentry et al,^15^ 2017	Characterize the informed consent discussion.	Audio-recording of consent conversations with subsequent coding and quantification of specific elements; Survey data evaluating demographic characteristics and subjective satisfaction levels.	Overall, 95% of informed consent conversations included some discussion of risk, and 70% contained ≥3 elements of informed consent (raw data not provided). Among subset of discussions that included ≥3 elements of informed consent, parental recall rates for risks, benefits, and anesthetic plan were 84%, 85%, and 97%, respectively (raw data not provided). Self-reported parental comprehension rates for these elements was 88%, 96%, and 96%, respectively (raw data not provided).	Discussion of uncertainty (48%) and discussion of patient preferences (18%) were most commonly missing elements of informed consent.	No data.	Parental recall of elements of informed consent was correlated with presence of ≥3elements of informed consent in preoperative discussions (ie, risks, benefits, and plan). Most parents (85%, raw data not provided) were satisfied with informed consent conversations, regardless of elements included in consent process.
Kindler et al,^16^ 2005	Describe the nature of the patient-anesthetist interaction and shared decision-making.	Utterances from the patient and anesthetist were coded using 2 previously validated coding tools; OPTION scores.	No specific data on discussion of risks. Mean of 23% of utterances about counselling; this included 18.7% of utterances about describing various anesthetic techniques. Based on details of utterance codes, only 8.9% of utterances included discussion of benefits/risks of various anesthetic techniques. The remainder of the counselling utterances were about patient preparation (explaining techniques/logistics and expectation management) and patient reassurance.	In the 21 consultations that involved shared decision-making, mean OPTION scores were 26.8 (of 100). Anesthesia professionals commonly listed choices for anesthetic techniques (19 of 21 visits) but rarely confirmed patient understanding (2 of 21 visits). In addition, elicitation of patient expectations, concerns, and preferences was rare in OPTION scores.	Utterances about postoperative care were rare (2.3% of all utterances, including utterances about pain control).	Overall, 26% of utterances by physicians were questions, the minority of which were open ended (3.4%). Few utterances about psychosocial issues (<0.1%) or empathizing (0.5%). Statistically significant associations were found between use of open-ended questions, facilitating statements, and emotional statements by anesthetists and level of patient involvement, but the magnitude of these correlations was small.
Lagana et al,^17^ 2012	Observe and identify the number and nature of anesthesia risks considered and communicated to parents/guardians.	No. of risks discussed in preoperative interviews.	27/91 consultations contained no discussion of risk. 23/91 consultations only included general statements with no elaboration or discussion of material consequences. Most common risks discussed: nausea and vomiting (36%); sore throat (35%); allergy (29%); hypoxia (25%); and emergence delirium (19%).	No data.	Specific situations reflecting care beyond the OR rarely mentioned (prolonged intubation, 1 instance; prolonged admission, 3 instances; death, 0 instances).	No additional data.
Nuebling et al,^18^ 2004	Observe the association between physicians’ reassuring utterances and a variety of patient utterances.	Audio recording of patient encounters with coding and categorization of utterances from patients and physicians.	No data.	No data.	No data.	Physician reassuring and/or optimistic utterances were associated with patient utterances that asked for reassurances, expressed concern, or expressed optimism and/or self-reassurance.
Sandberg et al,^19^ 2008	To quantify the amount of information given to patients by anesthesia clinicians during preanesthetic interviews.	Audio recordings of preanesthetic consultations with quantification of volume of information conveyed during encounters.	No. of information units in professionals’ communication greatly exceeded patients’ information storing capacity. Nurses provided more informational units compared with physicians (mean [SD], 112 [37] vs 49 [25]; *P* < .001) Overwhelming majority of communication behavior related to informational units.	No data.	No data.	No additional data.
Stubenrouch et al,^20^ 2017	Determine the level of shared decision-making in anesthesia consultations.	Audio recording of preanesthetic encounters with subsequent coding of transcripts; OPTION scores; Survey data using SDM-Q-9 with patients and anesthesiologists.	Health care professionals rarely explain benefits and risk of various anesthetic options.	Health care professional do not explain the need to deliberate and/or consider anesthetic options in conjunction with patient preferences, infrequently elicit patient preferences, and do not make adequate attempts to integrate patient preferences into decision-making.	No data.	Perception of quality of shared decision-making high among clinicians and patients, as measured by SDM-Q-9 and SDM-Q-Doc, despite objective measures of adequacy of shared decision-making being very low.
Tait et al,^21^ 2011	To examine the information that parents sought regarding their child’s anesthesia, what they are told, who told them, and how much of the information they could recall.	Semistructured interviews assessing parental recall and comprehension of information in preanesthetic consultations and parental preferences for how to receive information; Timing of consent and role of person obtaining consent.	Overall, 96% of parents recalled a description of anesthesia at the time of consent, and 81% recalled a discussion about postoperative pain control. More than half could recall a discussion about risks and benefits of anesthesia; 46.2% of parents reported having a complete understanding of anesthesia, and 42.4% reported having a complete understanding of pain management. Very few (11%) reported having a complete understanding of risks and benefits of anesthesia.	No data.	Postoperative pain control recalled in 81% of cases (no raw data), but complete understanding only in 101/238 follow-up interviews (42.4%).	Most parents preferred a combination of written and verbal information during the consent process, and most preferred to have consent done within a week of surgery. Parental recall appeared to be better when consent was taken on the day of surgery and when consent was taken by an anesthesia professional.
Trumble et al,^22^ 2015	To describe and quantify the risks and benefits of epidural anesthesia during the consent process	Audio recording of consent conversations prior to epidural placement with coding and quantification of risks discussed.	No. of risks discussed prior to procedure varied from 0 to 11, with a median of 7 risks per discussion. The most commonly discussed risks were failed block, postdural puncture headache, nerve damage, epidural bleeding, and epidural infection. At least 1 risk was quantified in 71.4% of discussions. Benefits and alternatives were discussed in only 21.4% of cases.	No data.	No data.	No data.
Zollo et al,^23^ 2009	To observe and describe the patterns of communication in the preanesthesia clinic with 2 types of standardized patients (ie, information seeker and information blunter).	Audio recording of encounters with standardized patients with quantification of time spent on various aspects of the interview; Postencounter questionnaires and patient satisfaction scores.	Mean of 2 min spent discussing risks, with slightly more time spent when patient is an information seeker vs information blunter (1.6 min vs 2.4 min). Specific risks discussed varied by encounter. No specific data on counseling, reassurance, or preparation. Mean of <1 min spent making a plan, 2-3 min spent describing a general anesthetic, and 1.2 min discussing postoperative pain control.	Overall, <1 min spent obtaining patient perspective in preoperative encounters. In postinterview patient questionnaires, responses to the questions, “To what extent did the anesthetist ask about your goals for the anesthetic and recovery?” and “To what extent did the anesthetist encourage you to take the role you wanted in your own care” were mostly “a little” or “not at all,” indicating infrequent elicitation of patient preferences.	Mean of 1.2 min discussing postoperative pain control. No data on discussion of broader postoperative care.	Improved satisfaction scores with more experienced anesthesia professionals in the information seeker interviews and with those who reported having previously taken any kind of communications course.

Abbreviations: OPTION, Observing Patient Involvement Scores; SDM-Q-9, 9-item Shared Decision-Making Questionnaire.

#### Informed Consent and Patient Comprehension

Ten studies^12,13,15,16,17,19,20,21,22,23^ included data on these topics. Two studies^16,23^ examined communication in general without a specific focus. One study of patient and anesthesiologist utterances during consultations^16^ identified a mean of 23% of utterances as being related to patient counseling (exact proportion not provided); however, the coding method used suggests that most utterances coded as counseling were likely related to technical and logistical aspects of care. A similar result was seen in a study of anesthesia consultations with standardized patients,^23^ which used mock patient scenarios and 2 different standardized patients. In this study, only a mean of less than 1 minute was spent making a plan in each encounter.^23^

Seven studies^13,15,16,17,20,22,23^ contained data about risk and benefit discussions; 3 studies^13,17,22^ specifically evaluated communication of risks. One study^17^ found that during 91 clinical encounters with parents of children undergoing anesthesia, in 27 consultations (29.6%) no risks were discussed, and in a further 23 consultations (25.3%), only a general statement of risk was included. Serious risks were only discussed in 4 encounters (4.4%). In adults undergoing elective surgery, another study^13^ found that during 40 routine encounters, only 31 preoperative consultations (77.5%) included discussion of at least 1 risk. Where risk was part of consultations (n = 151), patients were almost always satisfied and not distressed by the discussion (146 of 151 consultations [96.7%]). Conversely, in consultations where no risks were discussed (n = 115), most patients (96 [83.5%]) believed that there was no risk to anesthesia at all.^13^ A small study on epidural insertion^22^ found a similar degree of variability, where the number of risks discussed in consent conversations varied from 0 to 11 per encounter. In studies with a focus other than risk communication that had ancillary data about risk discussions, there was a similar degree of variability.^15,16,20,23^ Most risks specifically evaluated in these studies were minor, short-term risks. Global assessment of informed consent was evaluated in only 1 study,^15^ which found that in conversations with parents of children undergoing anesthesia, the minimum requirements for informed consent were included in 68 of 97 cases (70.1%). Only a minority of conversations (12%, exact proportion not provided) included all 7 aspects of fully informed consent as defined by the authors.

Data related to patient comprehension of information communicated by anesthesiologists were extracted from 4 studies.^12,15,19,21^ Among studies with objective measures of patient comprehension, patient understanding of risks and benefits of various anesthetic options was poor. For example, 1 study showed that many parents recalled a description of the anesthesia planned for their child (96.2%, exact proportion not provided) and plans for postoperative pain control (81.2%, exact proportion not provided), but follow-up questions suggested very few parents fully understood risks, benefits, and complications (28 of 263 parents [10.6%]).^21^ In another study, parents frequently reported understanding risks, benefits, and the anesthetic plan (88%, 96%, and 96%, respectively; exact proportions not provided).^15^ However, this study only included self-reported parental comprehension. When considering specific words used in consultations, 1 study^12^ demonstrated that although patients misunderstood a minority of technical terms used by anesthesiologists (49 of 484 terms [10.1%] misunderstood across all encounters), there was at least 1 instance of patients misunderstanding in 32 of 68 individual encounters (47.1%).^12^ Another aspect of communication relating to patient comprehension was evaluated by a study that measured the amount of information given to patients preoperatively by anesthesia professionals.^19^ This study found that patients’ information storing capacity was consistently exceeded in preoperative encounters.^19^

#### Shared Decision-Making

Five studies^14,15,16,20,23^ had data about eliciting patient preferences and shared decision-making. In the 2 studies^16,23^ that evaluated communication generally, elicitation of patient preferences and values was uncommon. In 1 study,^23^ anesthesiologists spent less than 1 minute obtaining patient perspectives during encounters that were a mean (SD) of 15.9 (4.9) minutes long. Another study^16^ showed no utterances eliciting patient preferences during consultations. Across 21 encounters in this study that required a shared decision, the Observing Patient Involvement Scores (OPTION scores^25^) were poor, with elicitation of patient input categories receiving the lowest scores.^16^ There were similar findings in a study of informed consent in pediatric anesthesia,^15^ which showed that elicitation of parental preferences was uncommon (18% of consultations, exact proportion not provided). Two studies^20,14^ examined shared decision-making. In 1 study of shared decision about neuraxial vs general anesthesia,^20^ OPTION scores showed that anesthesia professionals rarely explained the benefits and risks of anesthetic options and did not elicit or make adequate attempts to integrate patient preferences into decision-making. Another study^14^ had similar findings: most patients (>90%) wanted to be involved in decisions about their care, and anesthetists tended to underestimate patients’ desire for shared decision-making.

#### Discussions About Postoperative Care

Discussions about postoperative care were rare: this type of communication was described in 5 studies,^13,16,17,21,23^ and postoperative pain control dominated these discussions. Only 2 studies^13,17^ presented data on communication about specific adverse outcomes. In these studies, there were 4 instances of communication about postoperative events across 91 interviews (4.4%) in 1 study,^17^ and death or severe permanent harm discussed in 20 of 272 interviews (7.4%) and 22 interviews (8.1%), respectively, in another study.^13^ None of the studies had any data about elicitation of patient preferences regarding direction of care in the case of serious adverse events.

#### Other Data

Eight studies^13,14,15,16,18,20,21,23^ had some additional data about patient satisfaction or perception of the quality of the encounter following anesthesia consultations. Satisfaction was generally high, regardless of which specific components were included in interviews,^15,20^ and satisfaction may have a positive association with degree of patient involvement in care decisions^14^ and with more experienced anesthesia professionals.^23^

## Discussion

This systematic review of the literature on communication between anesthesia professionals and patients found only 12 studies that met inclusion criteria. The studies had an overall moderate level of methodological quality. The main finding is that communication about postoperative care was rarely described in preoperative consultations with anesthesia professionals; the literature had no data describing anesthesiologist-patient communication addressing protracted ICU stay, protracted ventilation, and end-of-life care in the setting of postoperative incomplete recovery. These findings are consistent with a previous narrative review on patient-anesthesiologist communication^7^; however, this review contributes a more robust summary of the evidence by using a systematic search strategy, extracting and qualitatively collating data from superior data sources, assessing the quality of each study using an established evaluation tool, and including 11 studies that, to our knowledge, have not been summarized in any previous review on this topic. These data are also similar to previously published data on surgeon communication, showing little elicitation of values or preferences regarding these issues in surgical consultations.^1,2^ Therefore, most patients who undergo major surgical interventions do not have preoperative discussions about values, preferences, or goals of care that address the scenario of protracted or incomplete recovery from surgery. Several other findings emerge from the data. First, informed consent, including discussion of risks and benefits, is highly variable, and patient comprehension of risks, benefits, and therapeutic alternatives is frequently poor when measured objectively. Second, anesthesia professionals frequently give patients unmanageable amounts of information, and communication is often focused on technical and logistical aspects of care. Lastly, anesthesiologists infrequently engage in elicitation of patient values and shared decision-making, despite patients’ apparent desire to be involved in decision-making.

Professional society guidelines in anesthesia recommend that “anesthesiologists should include patients, including minors, in medical decision making that is appropriate to their developmental capacity and the medical issues involved.”^26^ However, there are many barriers to discussions of patient values, preferences, and goals of care in the preoperative setting. In many North American surgical centers, anesthesiologists only become involved in the care of surgical patients after they have made the decision to proceed to the operating room with their surgeon. Therefore, it is likely that many anesthesiologists focus on getting the patient through the operation and may see this kind of communication and patient value exploration as not a part of their job. Second, there are large financial incentives to proceed to the operating room, for surgeons as well as anesthesiologists, putting additional emphasis on moving the patient through the operating room. Third, many anesthesiologists lack the specific expertise to speak to perioperative issues that reach beyond the operating room. Lastly, given the volume of patients seen at anesthetic clinics, anesthesiologists likely feel tremendous time pressure and probably feel they do not have adequate time for (and are not adequately compensated for) protracted discussion of perioperative values, preferences, and goals of care. In cases where there are nontrivial risks that may result in a significant change in a patient’s health status or prolonged burdensome care (for example, ventilator dependence after a postoperative stroke), then anesthesia consultations without discussion of postoperative care and elicitation of patient preferences may represent a missed opportunity to raise these issues. Identifying patients at risk for postoperative complications, such as prolonged mechanical ventilation, weakness, and postoperative delirium, can provide an important perspective on perioperative decisions. Literature on communication from outside anesthesiology suggests that patients often agree to a plan of care that is inconsistent with their values and preferences, including undergoing surgery.^27^ Informed consent for major surgery that explores these factors is often possible in a 20- to 30-minute clinical encounter.^28^ Therefore, it seems both feasible and valuable for anesthesiologists to engage in balancing risks and benefits in the context of the patient’s values during preoperative consultations, especially when anesthesiologists are involved in postoperative care.

The 2 other physician specialties that routinely encounter surgical patients, namely surgery and critical care, have studied communication extensively compared with the findings here. For example, there have been several recent reviews on surgeon-patient communication,^1,29,30^ including a systematic review^1^ that only included studies with audiotaped or videotaped interactions and at least 1 objective measure of surgeon behavior or communication skills. This review reported data from 21 studies and an additional 13 companion reports. If these selection criteria were applied to this systematic review of anesthesia and patient communication, only 1 study would be included. Similarly, there have been multiple reviews of physician-patient communication in critical care medicine,^31,32,33^ a discipline in which specific types of communication, such as end-of-life communication^34,35^ and communication strategies for difficult decision-making,^36,37,38^ have been broadly evaluated.

Based on the data in this review, several hypotheses follow regarding strategies to improve patient-anesthesiologist communication. First, if anesthesia professionals adapt to patients’ individual communication needs, patient participation and satisfaction may improve, although this strategy has not led to measurable improvement in communication about end-of-life issues in ICU.^39^ Second, while there is tension between providing too much information (risking information overload) and not providing enough information (risking inadequate patient understanding and informed consent), the data suggest that communication may improve if anesthesia professionals identify and emphasize important nontechnical information specific to each individual patient. Lastly, for anesthesiologists involved in perioperative medicine, patients who are at high risk of incomplete recovery may benefit from elicitation of values and preferences regarding postoperative care during preoperative consultations. Shifting the focus of anesthetic care to perioperative medicine and specifically improving preoperative communication about goals of care is likely to be a significant challenge for the specialty of anesthesiology. Several interventions aimed at perioperative advance care planning have been developed and evaluated,^40,41,42^ providing some guidance for anesthesiologists expanding their practice into perioperative medicine.

### Limitations

This systematic review has several limitations. First, the search was limited to studies published in English from 1980 to April 2020. Although additional data may have been published earlier or indexed elsewhere, they are not likely to be relevant to current practice. The search only found 12 studies with different designs, settings, and outcomes, making synthesis challenging. Common limitations for the studies that were reviewed included unavoidable selection bias due to selective participation; the Hawthorne effect in studies that employed direct observation (2 of 12 studies), and the infrequent use of validated analysis or coding tools (only 4 of 12 studies used validated tools). The survey-based studies (3 of 12 studies) were limited by recall bias of patients and health care professionals. Nine studies implemented mitigation strategies for these biases. Lastly, only 3 studies provided data about the risk category of the patients in their analyses, and most patients were considered low risk for complications. Preoperative communication with patients with higher risk may be substantially different compared with the communication patterns found in this review. These limitations make it difficult to draw concrete conclusions about communication in anesthesia and implications for patients who have incomplete recovery.

## Conclusions

This systematic review of the literature on patient-anesthesiologist communication found that communication in anesthesia rarely includes discussion of postoperative care or patient values and preferences, but rather is dominated by anesthetic planning and perioperative logistics. These findings, coupled with similar data from surgical literature, suggest that most patients who arrive in the critical care unit following a major operation have not had a preoperative discussion about values, preferences, and goals of care specific to protracted recovery or prolonged intensive care.

## Notes

Supplement. **eAppendix 1. **MEDLINE Search Strategy **eAppendix 2. **Study Inclusion Criteria **eAppendix 3. **Study Exclusion Criteria **eAppendix 4. **Studies Excluded on Full Text Review
