# Supplementary material for: Anesthesiologist to Patient Communication: A Systematic Review
Source: JAMA Netw Open. 2020 Nov 12;3(11):e2023503. doi: 10.1001/jamanetworkopen.2020.23503 (PMC7662141; doi:10.1001/jamanetworkopen.2020.23503)
Supplement: Supplement. — eAppendix 1. MEDLINE Search Strategy eAppendix 2. Study Inclusion Criteria eAppendix 3. Study Exclusion Criteria eAppendix 4. Studies Excluded on Full Text Review [file jamanetwopen-e2023503-s001.pdf]

Supplemental Online Content

Tylee MJ, Rubenfeld GD, Wijeysundera D, Sklar MC, Hussain S, Adhikari NKJ. Anesthesiologist to patient communication: a systematic review. *JAMA Netw Open*. 2020;3(11):e2023503. doi:10.1001/jamanetworkopen.2020.23503

eAppendix 1. MEDLINE Search Strategy
eAppendix 2. Study Inclusion Criteria
eAppendix 3. Study Exclusion Criteria
eAppendix 4. Studies Excluded on Full Text Review

This supplemental material has been provided by the authors to give readers additional information about their work.

© 2020 Tylee MJ et al. *JAMA Network Open.*

eAppendix 1. MEDLINE Search Strategy (*number of articles found with each search string shown in brackets*)

1. exp Anesthesia/ or exp Anesthesiology/ (205581)
2. (anesthesiologist or anesthesiologist).mp. [mp=title, abstract, original title, name of substance word, subject heading word, keyword heading word, protocol supplementary concept word, rare disease supplementary concept word, unique identifier] (6232)
3. exp Nurse Anesthetists/ or anesthetist.mp. or anesthetist.mp. (4237)
4. exp Communication/ (305316)
5. Physician-Patient Relations/ (72124)
6. exp Patient Education as Topic/ (84926)
7. (patient adj3 prepar*).mp. (4041)
8. (patient adj5 (physician or doctor) adj5 communicat*).mp. (4828)
9. exp Informed Consent/ (40859)
10. (1 or 2 or 3) and (4 or 5 or 6 or 7 or 8 or 9) (3316)

© 2020 Tylee MJ et al. *JAMA Network Open.*

eAppendix 2. Study Inclusion Criteria

1. Study undertaken with the *primary intent* of exploring, describing, or quantifying the content and patterns of communication between anesthesia providers (CRNAs or Anesthesiologists) and patients.

© 2020 Tylee MJ et al. *JAMA Network Open.*

eAppendix 3. Study Exclusion Criteria

1. Study does not examine communication as the primary focus (for example, a study that examines pre-admission clinic patient encounters, but is focused on patient flow or other hospital efficiencies, as opposed to communication)
2. Study examines physician communication with individuals or groups who are not patients (for example, a study with data on physician-physician communication or physician-nurse communication)
3. Study does not examine anesthesiologist communication (for example, a study on surgeon-patient communication)
4. Study does not have original data (for example, opinion pieces)
5. Study only looks at provider perception of their communication in general (that is, the raw data is only provider reflections on their practice in general, as opposed to data about the content of specific patient-physician interactions)
6. Study examines communication at the time of anesthetic procedure (such as epidural catheter insertion or nerve blockade).
7. Study primarily examines the effect of adjuncts to communication, such as information booklets or videos, with no data about communication patterns or content with an anesthesia provider
8. Study is about communication with pediatric patients
9. Study is about communication during recruitment into anesthesia research studies
10. Study was published before 1980
11. Study is about patient perceptions of anesthesiologists or narratives about anesthesia, not about communication

© 2020 Tylee MJ et al. *JAMA Network Open.*

eAppendix 4. Studies Excluded on Full Text Review

1. Clark SK, Leighton BL, Seltzer JL. A risk-specific anesthesia consent form may hinder the informed consent process. Journal of Clinical Anesthesia 1991; 3:11–3
2. DeMaria SJ, DeMaria AP, Silvay G, Flynn BC. Use of the BATHE method in the preanesthetic clinic visit. Anesthesia & Analgesia 2011; 113:1020–6
3. Elsass P, Eikard B, Junge J, Lykke J, Staun P, Feldt-Rasmussen M. Psychological effect of detailed preanesthetic information. Acta Anesthesiol Scand 1987; 31:579–83
4. Granziera E, Guglieri I, Del Bianco P, Capovilla E, Dona' B, Ciccarese AA, Kilmartin D, Manfredi V, De Salvo GL. A multidisciplinary approach to improve preoperative understanding and reduce anxiety: a randomised study. Journal of Anesthesiology 2013; 30:734–42
5. Harms C, Young JR, Amsler F, Zettler C, Scheidegger D, Kindler CH. Improving anesthetists' communication skills. Anesthesia 2004; 59:166–72
6. Inglis S, Farnill D. The effects of providing preoperative statistical anesthetic-risk information. Anesthesia & Intensive Care 1993; 21:799–805
7. Kain ZN, Wang SM, Caramico LA, Hofstadter M, Mayes LC. Parental desire for perioperative information and informed consent: a two-phase study. Anesthesia & Analgesia 1997; 84:299–306
8. Lilja Y, Ryden S, Fridlund B. Effects of extended preoperative information on perioperative stress: an anesthetic nurse intervention for patients with breast cancer and total hip replacement. Intensive & Critical Care Nursing 1998; 14:276–82
9. Soltner C, Giquello JA, Monrigal-Martin C, Beydon L. Continuous care and empathic anesthesiologist attitude in the preoperative period: impact on patient anxiety and satisfaction. Journal of Anesthesia 2011; 106:680–6
10. Carnie J. Patient feedback on the anesthetist's performance during the pre-operative visit. Anesthesia 2002; 57:697–701
11. Cheng WYC, Blum P, Spain B. Barriers to effective perioperative communication in indigenous Australians: an audit of progress since 1996. Anesthesia & Intensive Care 2004; 32:542–7
12. Clifton PJ. Expectations and experiences of anesthesia in a District General Hospital. Anesthesia 1984; 39:281–5
13. El-Sayeh S, Lavies NG. Pre-operative information about anesthesia--is more better? Anesthesia 2003; 58:1119–20
14. Gillies A, Gillies R, Weinberg L. Patient recollections of perioperative anesthesia risks. Anesthesia & Intensive Care 2013; 41:247–50

© 2020 Tylee MJ et al. *JAMA Network Open.*

15. Lam E, Lee M, Brull R, Wong DT. Effect of anesthesia consultation on patients' preoperative concerns. Journal of Anesthesia 2007; 54:852–3

16. Puro H, Pakarinen P, Korttila K, Tallgren M. Verbal information about anesthesia before scheduled surgery - contents and patient satisfaction. Patient Education & Counseling 2013; 90:367–71

17. Berg K, Kaspersen R, Unby C, Hollman Frisman G. The interaction between the patient and nurse anesthetist immediately before elective coronary artery bypass surgery. J Perianesth Nurs 2013; 28:283–90

18. Smith AF, Pope C, Goodwin D, Mort M. Communication between anesthesiologists, patients and the anesthesia team: a descriptive study of induction and emergence. Journal of Anesthesia 2005; 52:915–20

19. Slater P, Sellors J, Cyna AM. Communications during epidural catheter placement for labour analgesia. Anesthesia 2011; 66:1006–11

20. Rai E, Chen RY, Noi CS, Hee, HI. Evaluation of anesthesia informed consent in pediatric practice – An observation cohort study. Journal of Anaesthesiology Clinical Pharmacology 2019; 35: 515-21.

© 2020 Tylee MJ et al. *JAMA Network Open.*
